# IA-PACS-CFS: a double-blinded, randomized, sham-controlled, exploratory trial of immunoadsorption in patients with chronic fatigue syndrome (CFS) including patients with post-acute COVID-19 CFS (PACS-CFS)

**DOI:** 10.1186/s13063-024-07982-5

**Published:** 2024-03-07

**Authors:** Hannah Preßler, Marie-Luise Machule, Friederike Ufer, Isabel Bünger, Lucie Yuanting Li, Emilie Buchholz, Claudia Werner, Esther Beraha, Frank Wagner, Matthes Metz, Susen Burock, Lisa Bruckert, Christiana Franke, Nicola Wilck, Anne Krüger, Alexander Reshetnik, Kai-Uwe Eckardt, Matthias Endres, Harald Prüss

**Affiliations:** 1 Department of Neurology and Experimental Neurology, Charité-Universitätsmedizin Berlin, corporate member of Freie Universität Berlin, Humboldt-Universität zu Berlin and Berlin Institute of Health, Charitéplatz 1, Berlin, 10117 Germany; 2 Excellence Cluster NeuroCure, Berlin, Germany; 3 German Center for Neurodegenerative Diseases (DZNE) Berlin, Berlin, Germany; 4 Clinical Research Organisation GmbH, Charitéplatz 1, Berlin, 10117 Germany; 5 Department of Biostatistics, GCP-Service International Ltd. & Co. KG, Bremen, Germany; 6 Clinical Trial Office, Charité – Universitätsmedizin Berlin, Charitéplatz 1, Berlin, 10117 Germany; 7 Center for Stroke Research Berlin, Charité-Universitätsmedizin Berlin, Berlin, Germany; 8 Department of Nephrology and Medical Intensive Care Medicine, Charité-Universitätsmedizin Berlin, Berlin, Germany; 9 Department of Nephrology and Hypertension, Friedrich-Alexander-Universität Erlangen-Nürnberg, Erlangen, Germany; 10 Experimental and Clinical Research Center (ECRC), a cooperation of Charité - Universitätsmedizin Berlin and Max Delbruck Center for Molecular Medicine (MDC), Berlin, 13125 Germany; 11 Max Delbrück Center for Molecular Medicine in the Helmholtz Association (MDC), Berlin, 13125 Germany

**Keywords:** Chronic fatigue syndrome, Myalgic encephalomyelitis, Post-acute COVID-19 syndrome, Long-COVID, Immunoadsorption, Autoimmunity, PROMIS, Biomarker

## Abstract

**Background:**

Myalgic encephalomyelitis/chronic fatigue syndrome (ME/CFS) is a severely debilitating condition which markedly restricts activity and function of affected people. Since the beginning of the COVID-19 pandemic ME/CFS related to post-acute COVID-19 syndrome (PACS) can be diagnosed in a subset of patients presenting with persistent fatigue 6 months after a mostly mild SARS-CoV-2 infection by fulfillment of the Canadian Consensus Criteria (CCC 2003). Induction of autoimmunity after viral infection is a mechanism under intensive investigation. In patients with ME/CFS, autoantibodies against thyreoperoxidase (TPO), beta-adrenergic receptors (ß2AR), and muscarinic acetylcholine receptors (MAR) are frequently found, and there is evidence for effectiveness of immunomodulation with B cell depleting therapy, cyclophosphamide, or intravenous immunoglobulins (IVIG). Preliminary studies on the treatment of ME/CFS patients with immunoadsorption (IA), an apheresis that removes antibodies from plasma, suggest clinical improvement. However, evidence from placebo-controlled trials is currently missing.

**Methods:**

In this double-blinded, randomized, sham-controlled, exploratory trial the therapeutic effect of five cycles of IA every other day in patients with ME/CFS, including patients with post-acute COVID-19 chronic fatigue syndrome (PACS-CFS), will be evaluated using the validated Chalder Fatigue Scale, a patient-reported outcome measurement. A total of 66 patients will be randomized at a 2:1 ratio: 44 patients will receive IA (active treatment group) and 22 patients will receive a sham apheresis (control group). Moreover, safety, tolerability, and the effect of IA on patient-reported outcome parameters, biomarker-related objectives, cognitive outcome measurements, and physical parameters will be assessed. Patients will be hospitalized at the clinical site from day 1 to day 10 to receive five IA treatments and medical visits. Four follow-up visits (including two visits at site and two visits via telephone call) at month 1 (day 30), 2 (day 60), 4 (day 120), and 6 (day 180; EOS, end of study visit) will take place.

**Discussion:**

Although ME/CFS including PACS-CFS causes an immense individual, social, and economic burden, we lack efficient therapeutic options. The present study aims to investigate the efficacy of immunoadsorption and to contribute to the etiological understanding and establishment of diagnostic tools for ME/CFS.

**Trial registration:**

Registration Number: NCT05710770. Registered on 02 February 2023.

## Administrative information

Note: the numbers in curly brackets in this protocol refer to SPIRIT checklist item numbers. The order of the items has been modified to group similar items (see http://www.equator-network.org/reporting-guidelines/spirit-2013-statement-defining-standard-protocol-items-for-clinical-trials/).

**Table:** 

Title {1}	IA-PACS-CFS: A double-blinded, randomized, sham-controlled, exploratory trial of immunoadsorption (IA) in patients with chronic fatigue syndrome (CFS) including patients with Post-acute COVID-19 CFS (PACS-CFS).
Trial registration {2a and 2b}.	Study is registered in ClinicalTrials.gov. Registration Number: NCT05710770. Registered on 02 February 2023. https://classic.clinicaltrials.gov/ct2/show/NCT05710770
Protocol version {3}	Version 3.1 Including Amendment 3 / JUN 2023
Funding {4}	The study is supported by funds from the German Federal Ministry of Education and Research (grant 01EP2201). In addition, there is co-funding from Miltenyi Biotec company., which is involved in providing the apheresis material. Besides, Miltenyi contributed to covering the costs of financing a physician position.
Author details {5a}	Hannah Preßler^1,2#^, Marie-Luise Machule^1,3#^, Friederike Ufer^1,3^, Isabel Bünger^1,3^, Lucie Yuanting Li^1,3^, Emilie Buchholz^1,3^, Claudia Werner^4^, Esther Beraha^4^, Frank Wagner^4^, Matthes Metz^5^ Susen Burock^6^, Lisa Bruckert^6^, Christiana Franke^1,3^, Nicola Wilck^8,10,11^, Anne Krüger^8^, Alexander Reshetnik^8^, Kai-Uwe Eckardt^8,9^, Matthias Endres^1,2,3,7^, Harald Prüss^1,3^* ^1^ Department of Neurology and Experimental Neurology, Charité-Universitätsmedizin Berlin, corporate member of Freie Universität Berlin, Humboldt-Universität zu Berlin and Berlin Institute of Health, Berlin Germany ^2^ Excellence Cluster NeuroCure Berlin Germany ^3^ German Center for Neurodegenerative Diseases (DZNE) Berlin, Berlin, Germany ^4^ Clinical Research Organisation GmbH, Charitéplatz 1, 10117 Berlin, Germany ^5^ Department of Biostatistics, GCP-Service International Ltd. & Co. KG, Bremen, Germany ^6^ Clinical Trial Office, Charité – Universitätsmedizin Berlin, Charitéplatz 1, 10117 Berlin ^7^ Center for Stroke Research Berlin, Charité-Universitätsmedizin Berlin, Germany ^8^ Department of Nephrology and Medical Intensive Care Medicine, Charité Universitätsmedizin Berlin ^9^ Department of Nephrology and Hypertension, Friedrich-Alexander-Universität Erlangen-Nürnberg, Erlangen, Germany. ^10^ Experimental and Clinical Research Center (ECRC), a cooperation of Charité - Universitätsmedizin Berlin and Max Delbruck Center for Molecular Medicine (MDC), 13125 Berlin, Germany ^11^ Max Delbrück Center for Molecular Medicine in the Helmholtz Association (MDC), 13125 Berlin, Germany ^#^ These authors contributed equally. * Corresponding author: Harald Prüss, MD, Charité-Universitätsmedizin Berlin, Department of Neurology with Experimental Neurology, Charitéplatz 1, 10117 Berlin, Germany, Phone: + 49 450 660284, Email: harald.pruess@charite.de
Name and contact information for the trial sponsor {5b}	Charité Universitätsmedizin Berlin, corporate member of Freie Universität Berlin, Humboldt-Universität zu Berlin and Berlin Institute of Health, Berlin Germany, Sponsor Delegated Person: Prof. Dr. med. Matthias Endres Medical Director of the Charité Centre 15 for Neurology, Neurosurgery und Psychiatry (CC15), Charité Universitätsmedizin Berlin Charitéplatz 1 10117 Berlin, Germany Phone: + 49-30 450 560101 Email: matthias.endres@charite.de
Role of sponsor {5c}	IA-PACS-CFS is an investigator-initiated trial. The financial sponsors (German Federal Ministry of Education and Research and Miltenyi Biotec) had no role in the study design and will not have any role in collection, management, analysis, and interpretation of data, writing and submission of the study report for publication. The legal sponsor Charité Universitätsmedizin Berlin with his sponsor-delegated person Prof. Matthias Endres and the IA-PACS-CFS study group consisting of the authors of the current publication, however, have ultimate authority over all the activities.

## Introduction

### Background and rationale {6a}

Myalgic encephalomyelitis/chronic fatigue syndrome (ME/CFS) is a severely debilitating condition with an estimated worldwide prevalence of 0.89% [1], which markedly restricts activity and function of patients. They experience chronic severe fatigue even after minimal exercise or mental activity, together with a myriad of further symptoms such as cognitive impairment, poor sleep quality, muscle and joint pain, or headache. With the pandemic situation, ME/CFS related to post-acute COVID-19 syndrome (PACS) is becoming a raising issue. According to the World Health Organization (WHO), there are more than 768 million confirmed cases of COVID-19 worldwide, and a large proportion of patients discharged from hospitals are experiencing persistent symptoms [2, 3]. In principle, long-COVID can be observed following both mild and severe trajectories of the acute infection. A recent epidemiological study has indicated a global prevalence of long-COVID at approximately 37% and 49% during the intervals of 30 and 120 days subsequent to the initial infection, respectively [4]. One study showed that approximately 45% of the patients presenting with persistent moderate to severe fatigue 6 months after a mostly mild SARS-CoV-2 infection fulfil the Canadian Consensus Criteria (CCC 2003) for ME/CFS [5, 6]. The etiology and the mechanisms leading to the typical chronic course of ME/CFS or the pathophysiology of PACS are not fully understood. Disease concepts range from neuroimmunological to psychosomatic explanations. The lack of diagnostic tools or other validated biomarkers impede the diagnostic accuracy. Several non-evaluated treatments are in use for ME/CFS, but until now, none has been approved by any competent health authority. In general, post-viral induction of autoimmunity can occur and has been observed after various types of viral infections. Several studies suggest the contribution of autoimmune mechanisms also in ME/CFS occurring after an infection. Autoantibodies against thyreoperoxidase (TPO), beta-adrenergic receptors (ß2AR), and muscarinic acetylcholine receptors (MAR) are frequently found in these patients [7]. Evidence for an autoimmune contribution to the pathogenesis of ME/CFS is supported in some studies showing the effectiveness of immunomodulatory therapies. Two studies showed therapeutic effect of B cell depleting monoclonal antibody rituximab, which led to a partial or complete remission in 60% of patients [8]. Another trial with the broadly immunosuppressive drug cyclophosphamide showed a beneficial effect [9]. Finally, various studies support the positive effect of human immunoglobulin treatment (IVIG) on fatigue symptoms and physical functioning especially of patients with severe ME/CFS [10, 11]. Recently, there is an accumulation of evidence that a post-viral autoimmune reaction with the presence of autoantibodies targeting different neuronal tissues is also involved in the pathogenesis of PACS-CFS [12, 13]. Autoantibodies against G-protein-coupled receptors (GPCR) such as ß2-adrenoreceptors and α1-adrenoreceptors, nociceptors, and angiotensin II-, muscarinic M_2_-, MAS-, and ET_A_-receptors were found in sera from patients with persistent long-COVID symptoms including fatigue [14]. Besides, in COVID-19 patients with neurologic symptoms, the presence of central nervous system reactive autoantibodies in the serum and cerebrospinal fluid (CSF) was shown [12, 15–17]. First studies on the treatment of ME/CFS patients with immunoadsorption (IA), an apheresis in which antibodies are removed from plasma, suggest clinical improvement in some patients [18, 19]. First insights for the use of extracorporeal apheresis in patients with PACS-CFS came from Bornstein et al. 2022, who reported significantly reduced levels of ß-adrenergic and muscarinic receptor autoantibodies as well as clinical improvement [13]. However, placebo-controlled trials investigating the effectiveness of IA in patients suffering from ME/CFS, including patients with PACS-CFS, are lacking. Considering the current amount of confirmed COVID-19 cases, we might soon be facing a huge number of chronically ill severely disabled patients, underlining the ethical and economic urgency to develop effective therapeutic options.

#### Research question

In the present RCT, the effect of IA—a well-tolerated, highly efficient gold standard treatment for diverse neurological autoimmune diseases—will be examined for the treatment of ME/CFS, including ME/CFS related to PACS-CFS. Assuming an autoimmune pathogenesis contributing to the disease, we hypothesize that enrolled patients will show significant clinical improvement after five treatments with IA compared to sham apheresis.

## Objectives {7}

### Primary objective

The primary objective of the trial is to explore the effectiveness of repeated immunoadsorptions (IA) in patients with ME/CFS, including patients with PACS-CFS.

### Secondary objectives

The aim of the secondary objectives is to investigate safety, tolerability, and the effect of IA on patient-reported outcome parameters, biomarker-related objectives, cognitive outcome measurements, and physical assessments.

### Trial design {8}

**Fig. 1:**
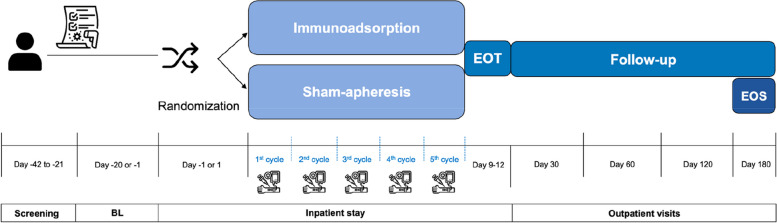
Schematic illustration of the study design. BL, baseline; EOT, end of treatment; EOS, end of study

This is a double-blinded, randomized, sham-controlled, exploratory trial to evaluate the therapeutic effect of five cycles of IA every other day in patients with ME/CFS, including patients with PACS-CFS. A total of 66 patients will be randomized at a 2:1 ratio: 44 patients will receive IA (active treatment group), and 22 patients will receive a sham apheresis (control group). Figure 1 gives a schematic overview of the study design.

During the screening for eligibility (max. 3 weeks (day − 42 to day − 21) and baseline assessment period, patients’ demographics, medical history, and blood and urine parameters (including routine blood and urine tests, viral serology, pregnancy, toxicology screening) will be assessed. In addition, cerebrospinal fluid and blood samples are taken to test for autoantibodies, markers of neurodegeneration, and neuroinflammation. Patient-reported outcome measurements, including Chalder Fatigue Scale, the Patient-Reported Outcomes Measurement Information System (PROMIS) questionnaire, and, in case of PACS-CFS, Post-COVID Functional Status (PCFS) Scale are performed either during the screening period or at the baseline visit. Physical assessments (finger tapping, handgrip strength evaluation, 6 Minute Walking Test (6MWT)), as well as autonomic assessments (such as the Schellong test), are carried out during either the screening or baseline visit. For cognitive assessment, we use the Montreal Cognitive Assessment (MoCA) as a widely recognized screening tool to detect mild cognitive impairments by assessing various cognitive domains such as attention and concentration, executive functions, memory, language, conceptual thinking, calculations, and orientation. Additionally, the Symbol Digit Modalities Test (SDMT) is a brief cognitive screening test that measures processing speed and attention, particularly useful in identifying cognitive deficits, where processing speed deficits may be evident. Both MoCA and SDMT are valuable tools to assess cognitive function and establish a baseline for monitoring any cognitive changes. At the baseline visit, optional spiroergometry and a detailed neuropsychological examination may be conducted.

Baseline assessments will be performed between days − 20 and − 1 and in any case before the first IA cycle. These include a general and neurological physical examination assessing vital signs, electrocardiography (ECG), and body weight, repeating safety check for pregnancy. Blood samples for chemistry, hematology, coagulation, and immunoglobulin titers are taken. Controlling for proximity of baseline measurements to the treatment schedule is imperative for safety reasons.

Patients are randomized to one of the two treatment arms between day − 1 and day 1. Peripheral venous access is preferred as vascular access for IA, and central venous access (Shaldon catheter) is established only if peripheral venous status is insufficient. Vascular access will thus be established before the first IA cycle on day 1 or day 2 (Shaldon catheter) or prior to each IA (peripheral venous access). Patients will be hospitalized at the clinical site from day 1 to day 9 or 10 and receive five IA treatments every other day and medical visits including monitoring of blood samples for chemistry, hematology, coagulation, and immunoglobulin titers every other day. Immunoadsorption will be performed using the LIFE21 apheresis unit and TheraSorb® Ig-omni 5 adsorber (Miltenyi Biotec), with treatment of two times the respective plasma volume per IA. To ensure blinding of patients, identical but IgG-saturated TheraSorb® Ig-omni 5 adsorbers will be used as sham procedure. Anticoagulation will be performed with heparin and citrate. While the IA team ensures the correct implementation of the respective active or sham treatment, details about study randomization will not be disclosed to patients or the rest of the study team.

Daily study visits are scheduled including control of vital signs and questioning for AE(s). After the last IA cycle, a blood sample for immunoglobulin titers is collected, the peripheral venous access (or Shaldon catheter) is removed, and patients are discharged from the hospital if the investigator has no concerns. At end-of-treatment visit (EOT), patients will also complete the Chalder Fatigue Scale, Bell Score, Physical-Function SF36, and Post-Exertional Malaise (PEM) questionnaires. If the IA treatments are performed according to the planned schedule, the EOT will take place on day 10. However, in the event of staffing limitations, resource constraints, or other factors that result in schedule changes, the EOT visit may vary between day 9 and 12. In addition, two of the four follow-up visits scheduled at month 2 (day 60) and month 6 (day 180; EOS, end of study visit) will take place at the neurological wards and outpatient clinics respectively. Patients will be asked by the study personnel to bring all requested questionnaires, which they completed in the meantime at home, to both follow-up visits, where further assessments and laboratory tests will be performed. An optional second cerebrospinal fluid sample may also be taken on day 60. The other two follow-up visits, scheduled at month 1 (day 30) and month 4 (day 120), will be conducted via telephone. During these visits, patients will be asked about any adverse events or concomitant medications, and questionnaires will be assessed. Patients will be followed up for a duration of 6 months, regardless of whether they receive sham or active treatment. This time frame has been chosen with the intention of identifying possible individuals that demonstrate a much late response to immunoadsorption than initially expected.

**Fig. 2:**
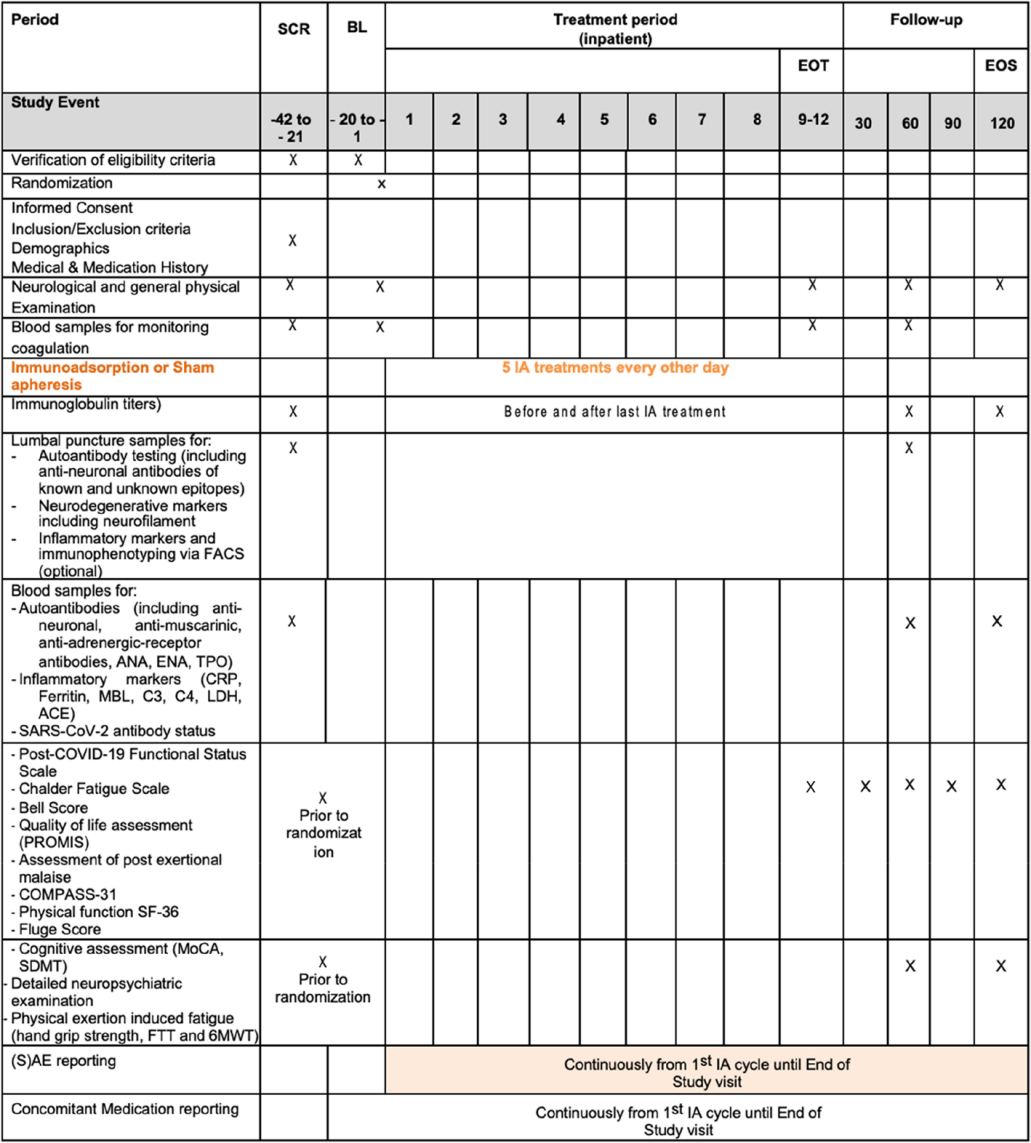
Schedule of assessments. Abbreviations: BL, baseline; CSF, cerebrospinal fluid; EOS, end of study visit; EOT, end-of-treatment visit; FACS, fluorescence-activated cell sorting; FTT, finger tapping test; IA, immunoadsorption; MoCA, Montreal Cognitive Assessment; 6 MWT, 6 Minute Walking Test; SDMT, Symbol Digit Modalities Test; PROMIS, Patient-Reported Outcomes Measurement Information System; (S)AE, (serious) adverse event, SCR, screening; SARS-CoV-2, severe acute respiratory syndrome coronavirus type 2

A more detailed overview is given in the schedule of assessments in Fig. 2.

## Methods: participants, interventions, and outcomes

### Study setting {9}

The study will take place at the three campuses of Charité-Universitätsmedizin Berlin, Germany. Patients will be recruited via the pPost-COVID-19 neurological outpatient clinic as well as the cChronic fFatigue immunological outpatient clinic. Patients will be hospitalized at the neurological inpatient clinics of the Charité, while IA and monitoring during the therapeutic intervention will be performed by the nephrology department at Charité.

### Eligibility criteria {10}

To participate in the study, patients must meet all inclusion criteria and no exclusion criterion.

The main inclusion criteria include fulfillment of the 2003 Canadian Consensus Criteria (CCC) for ME/CFS syndrome, experiencing prolonged post-exertional malaise, and having a Bell Score between 5 and 20. In addition, patients with PACS-CFS must provide a positive SARS-CoV-2 PCR test at least 6 months prior to screening to fulfil the subgroup criteria of the PACS-CFS. To identify patients exhibiting symptoms of ME/CFS prior to SARS-CoV-2 infection, we will carefully review patients’ medical records, specifically seeking evidence of chronic fatigue, post-exertional malaise, unexplained muscle pain, cognitive impairments, and other distinctive ME/CFS symptoms that strongly suggest pre-existing ME/CFS. Additionally, patients will undergo detailed questioning regarding their medical history, focusing one the cardinal symptoms that may have been present before SARS-CoV-2 infection, as well as any history of viral infections or tick-borne illness with similar symptoms, autoimmune disorders, or other conditions linked to the development of ME/CFS.

The main exclusion criteria are comorbidities bearing risk of not tolerating the treatment (judged by investigator), including malignant disease within the last 5 years, clinically meaningful laboratory abnormalities (e.g., renal insufficiency, cardiac insufficiency with a left ventricular ejection fraction (LVEF) lower than 40%, severe coronary heart disease, severe hypercoagulability; as well as acute or severe psychiatric disease, current indispensable medication with aAngiotensin-converting enzyme (ACE) inhibitors, fatigue duration for more than 5 years, presence of other conditions or differential diagnosis better explaining the symptoms of the patient than the suspected ME/CFS, ongoing immunosuppressive therapy and acute infectious diseases such as tuberculosis (TBC), human immunodeficiency virus (HIV), cytomegalovirus (CMV), Epstein-Barr vVirus (EBV), hepatitis B virus (HBV), and hepatitis C virus (HCV). The decision to exclude patients with a duration of fatigue exceeding 5 years from immunoadsorption treatment is based on the understanding that these individuals are more likely to have progressed to a chronic state of their condition, thereby limiting its effectiveness in restoring full functionality or alleviating symptoms.

### Who will take informed consent? {26a}

Prior to performing any study-related procedure, informed consent will be obtained from all trial participants. The investigator or a designated person will explain completely the aim of the project, the advantages and the risks of each strategy, handling of personal data, and the right of everyone to withdraw from the study. A corresponding written informed consent form will be provided and sufficient time to consider the participation is allowed.

### Additional consent provisions for collection and use of participant data and biological specimens {26b}

Variable response to study treatment may be due to genetic determinants that might affect disease etiology and/or molecular subtype of the disease. Therefore, an additional *voluntary* blood sample will be collected for DNA analysis from consenting participants for ancillary studies. Besides, patients will be asked to participate as well in the diagnostic platform of the National Clinical Study Group (NKSG), which is in line with other participating studies for harmonization purposes. The participation includes a *voluntary* cranial magnetic resonance tomography (cMRI) before and after IA-treatment as well as further blood samples for biomarkers.

## Interventions

### Explanation for the choice of comparators {6b}

In this study, a control group, receiving a sham IA (the adsorbers will be pre-saturated with immunoglobulins, so that it is therapeutic function of antibody removal is abolished) treatment arm is included. A sham IA is justified to minimize the potential bias in reporting of adverse events, assessing outcome measurements, and evaluating immunoglobulin levels and autoantibodies during this study. The 2:1 randomization ratio was chosen based on scientific evidence and clinical expertise, considering the high effectiveness of IA therapy in patients with other neuroimmunological diseases and the invasiveness of a sham-IA, ensuring sufficient statistical power in the active treatment arm.

### Intervention description {11a}

Two TheraSorb® Ig-omni 5 (Miltenyi Biotec) adsorbers are used for an IA procedure in conjunction with a tubing set, a plasma separator, and solutions for anticoagulation, regeneration, rinsing, and preservation. The procedure is controlled by an apheresis unit (LIFE 21, Miltenyi Biotec). Continuously withdrawn blood from a peripheral or central vein is anticoagulated and separated into its liquid and cellular components by plasma separation. The separated plasma is perfused through the first adsorber, where immunoadsorption to the adsorber matrix takes place. After a predefined volume of plasma has been processed by the first adsorber, the plasma is directed to the other adsorber, while the initially used adsorber is regenerated by elution of the previously bound target molecules. Consecutive switches between the two adsorbers are continued until the intended plasma volume has been processed. The immunoglobulin (Ig) depleted plasma is reunited with the previously separated blood cells and re-infused into the patient. After each treatment cycle, the patient is disconnected and the adsorber is rinsed with the preservation solution containing sodium azide and ethanol after final regeneration.

For IA therapy, the TheraSorb - Ig omni 5 adsorber (Miltenyi Biotec B.V. & Co. KG) will be used. The TheraSorb - Ig omni 5 adsorber is intended for the specific removal of human lambda and kappa chains containing immunoglobulins IgG (subclasses IgG1–IgG4), IgA, IgM, IgE, and immune complexes as well as free lambda and kappa light chains from human plasma in extracorporeal IA procedures. The TheraSorb - Ig omni 5 adsorber has a CE certification and is intended to be used for IA treatment of diseases where pathogenic immunoglobulins, lambda, or kappa light chains and immune complexes contribute to the onset of a disease or its progression. In case of sham apheresis, the adsorbers will be pre-saturated with immunoglobulins without adsorber regeneration during treatment, so that the therapeutic function in terms of antibody elimination is inhibited. If a Shaldon catheter is required, the insertion procedure is performed under sonographic control to prevent accidental puncture of carotid or pneumothorax. A chest X-ray is performed afterwards to ensure integrity of lungs. The implementation will take place either the day before or on the day of the first IA cycle. The number of IA cycles used is in accordance with earlier studies [18, 19]. IA is a well-established, generally safe interventional therapy broadly applied for different indications [20–22]. Medical complaints of the TheraSorb - Ig apheresis system are rare, and none of the medical complaints have resulted in long-term impairment of the patients’ health. The manufacturer’s internal data is presented in the Clinical Evaluation Report and the Post Market Clinical Follow-up Report. The Ig omni adsorbers specifically remove antibodies. Other components of the blood are not removed, but an unspecific loss of plasma is usually observed during the treatment. The TheraSorb - Ig omni adsorbers, which will be used in this study, with recombinant anti light chain antibody fragments as ligands, demonstrated a good performance in the clinical routine. So far, patients treated with the Ig omni adsorbers have tolerated the procedure very well. The rate of complaints with clinical symptoms of the patient was 0.03% after more than 12,000 treatments conducted. ACE-inhibitors will be paused at least 5 days prior to IA, since the adsorber may activate the bradykinin kallikrein system upon blood contact, and ACE-inhibitors can amplify this mechanism, leading to hypotension. Few cases of allergy like reactions (e.g., pruritus/itching of the skin, red skin, back pain, asthma) were observed with a frequency of about 1 case in 1000 treatments. In most treatments, the patients do not report any side effect. Blood pressure, pulse, and blood counts do not change significantly during the procedure. Electrolytes (phosphate and potassium) remain stable, but the blood levels of calcium may drop during the treatment due to the application of citric acid to prevent coagulation of blood in the apheresis unit. There may be temporary muscle cramps and discomfort in the arms and legs, mouth, and around the toes and fingers. However, there are no known interaction effects observed between the implemented anticoagulation measures and the further outcomes of interest in this study.

### Criteria for discontinuing or modifying allocated interventions {11b}

A patient will be discontinued from treatment in case of patient’s request, the occurrence of serious adverse events (SAEs) assessed as related or not related to study treatment, or an adverse event of severe intensity assessed as related to study treatment. Clinical adverse events, laboratory abnormalities, or intercurrent illness indicating that continued participation in the study would be harmful to the patient’s well-being will also lead to discontinuation. Additionally, any medical condition or clinically significant abnormal findings that may jeopardize the patient’s safety if study treatment is continued will also result in discontinuation. Regardless of the reason, patients will be asked to participate at follow-up assessments for safety reasons until 3 weeks after the last IA cycle (unless consent is withdrawn).

### Strategies to improve adherence to interventions {11c}

In a general perspective, we anticipate good compliance due to the absence of alternative therapies outside of potential studies. Additionally, our outpatient clinics provide extensive supervision, enabling us to foster motivation and readiness even after therapy completion. Patients will be educated on the significance of follow-up visits to assess the duration of potential clinical improvement with immunotherapy.

### Relevant concomitant care permitted or prohibited during the trial {11d}

The concomitant treatment with ACE-inhibitors during the intervention is prohibited, as this can lead to severe adverse events due to Bradykinin accumulation. Apart from that, there are no restrictions on activity during the study. To minimize post-exertional malaise (PEM) in ME/CFS, we recommend that all study patients use their own energy resources sparingly to strictly avoid overuse. Apart from oral corticosteroids (≤ 10 mg/day prednisone or equivalent) if on stable doses for at least 2 weeks prior to screening and during the entire study participation, other immunomodulatory therapies cannot be used 12 weeks before starting the IA treatment and during study participation. By including individuals using low-dose prednisone at a stable dosage in our study, we aim to prevent alterations in their baseline stability. The inclusion of participants with longstanding symptoms is facilitated by their access to a familiar and potentially beneficial medication, ensuring their willingness to engage in the study. It is important to underscore that the primary emphasis of the investigation remains on evaluating the impact of immunoadsorption, with the consideration of low-dose prednisone serving a supportive role in managing symptomatic relief.

### Provisions for post-trial care {30}

This is an approved and CE certified medical device, so we have no reason to believe that the study participants will suffer any permanent damage because of participating in the study. Clinical routine follow-ups after the end of the study will be provided by outpatient neurologists.

### Outcomes {12}

The primary outcome of the trial is the change in severity of tiredness in fatiguing illness, measured with the Chalder Fatigue Scale from baseline to 60 days post treatment. Clinical improvement will be quantified 2 months after completion of IA, using the change from baseline of the Chalder Fatigue Scale (Likert Score), a validated self-administered questionnaire to measure the severity of tiredness in fatiguing illnesses [23]. The Chalder Fatigue Scale has been used in multiple RCTs in patients with ME/CFS [24–27].

## Secondary outcomes

In terms of *safety-related* secondary outcomes, continuous surveillance of treatment emergent adverse events (TEAEs), serious adverse events (SAEs), and discontinuation due to TEAEs will be performed. Regarding *clinical effectiveness*, the secondary main outcomes include evaluation of sustained clinical improvement after completion of IA, using the Chalder Fatigue Scale at EOT (day 10), at months 1, 2, 4, and 6 after the completion of IA compared to baseline. Furthermore, effectiveness of IA regarding physical function and improvement in severity of CFS symptoms will be evaluated using the SF-36, Bell Score, Post-Exertional Malaise (PEM) questionnaire, and the Fluge Score (32 item questionnaire) at months 1, 2, 4, and 6 and using the repetitive hand grip strength test, finger tapping, and 6MWT at EOT (day 10) and at month 2 (and optional at month 6) after IA completion compared to baseline. Changes in functional outcome following COVID-19 using the PCFS scale will be documented at the same time points. Effect of IA on changes of autonomic function will be assessed using the Compass-31 questionnaire at months 1, 2, 4, and 6 after IA completion compared to baseline and by measuring blood pressure and heart rate regulation with Schellong test at EOT (day 10) and at 2 and optional 6 months after IA completion compared to baseline. In addition, effects of repeated IA on changes in neurocognitive functions performing a detailed neuropsychological examination in some cases as well as MoCA and SDMT will be evaluated in all patients at month 2 and at month 6 (if required by the investigator) after IA completion compared to baseline. Finally, the change in quality of life (QoL) using the PROMIS questionnaire is reported at month 2 and optional at month 6 after IA completion compared to baseline. In terms of *biomarker-related* secondary main outcomes, autoantibody titers against G-protein-coupled receptors (GPCR) such as ß2-adrenoreceptors and α1-adrenoreceptors, nociceptors, and angiotensin II-, muscarinic M_2_-, MAS-, and ET_A_-receptors, as well as autoantibodies against intracellular and surface antigens, immunoglobulin levels, and sub-types (IgA, IgM, IgG), inflammatory (CRP, ferritin, mannose binding lectin, white blood cell count, complement factors, cytokines), and neurodegenerative biomarkers including neurofilament and oligoclonal IgG bands, will be measured in blood at month 2 after IA completion compared to screening. Optional measurement in CSF will be conducted in case of positive findings in the screening. Please refer to Fig. 2 for the timepoint of each outcome.

### Participant timeline {13}

**Fig. 3:**
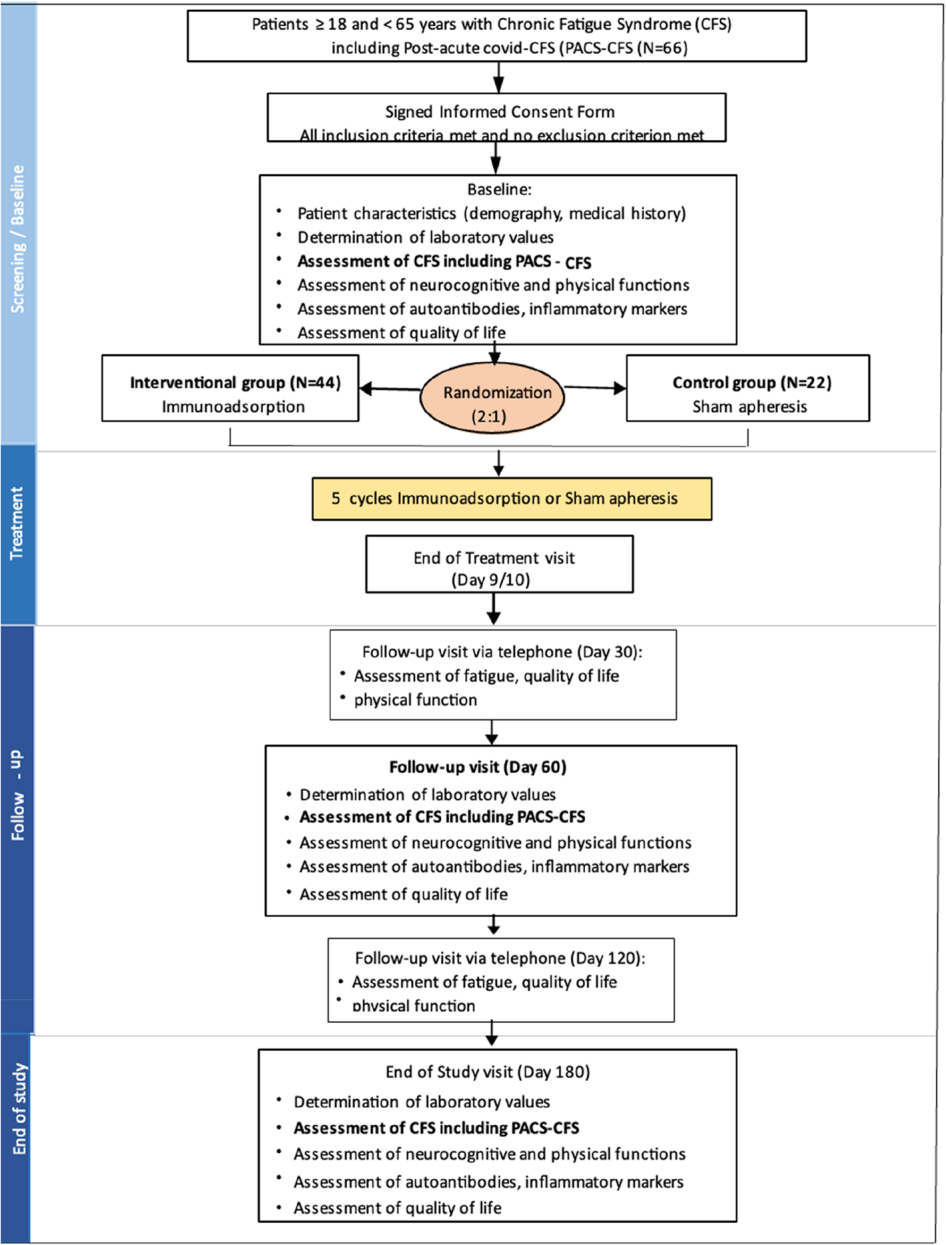
Study flow chart

The duration of the study will be up to 8 months (32 weeks) for each patient, including a 3-week screening period, a 10-day inpatient IA treatment, and a 6-month follow-up. This duration allows for the examination of the long-lasting effects of IA and provides an indication for the clinical feasibility of IA treatment in ME/CFS patients. The study flow chart is provided in Fig. 3.

### Sample size {14}

In this study, a total of 66 patients (allocated in a 2:1 ratio: IA group: 44 patients vs. sham group: 22 patients) will be included. Due to the exploratory nature of the study design, no formal statistical hypothesis testing is planned, and, therefore, no formal sample size calculation is performed. The expected potential information obtained from the current study is discussed below to guide the interpretation in relation to a clinically meaningful result. Limited data from two small pilot studies, investigating IA in 10 patients with ME/CFS, is available to date. Consequently, data is lacking about precise effects, clinical outcomes, and effect sizes in this group of patients. The justification of the sample size is based on the primary endpoint (change from baseline of the Chalder Fatigue Scale at day 60). The Chalder Fatigue Scale measured the fatigue in patient using the overall score (range from 0 to 33) derived from a questionnaire with 11 questions (each scored from 0 to 3, see Chalder 1993 [28]; Cella & Chalder [23] for more details). Based on a previous study [23], the mean score in a population of patients suffering from CFS is 24.4 (with a standard deviation of 5.8), and for a healthy sample, a mean value of 14.2 (with a standard deviation of 4.6) was observed. A difference of 10 points cannot be expected in this study where only patients suffering from CFS are included. In the systematic review of Åsa Nordin et al. 2016 [29], a minimal important difference for the global change ranges between 2.3 and 3.3. In an earlier study, examining the effectiveness of various treatments for ME/CFS, the difference in means of the Chalder Fatigue Scale was about 4 points (Likert score) when comparing the active to the control group among individuals diagnosed with ME/CFS [30]. However, no sham-controlled study evaluating the effect of IA on ME/CFS has been conducted so far, but first pilot studies suggest a positive effect of IA in patients with ME/CFS. To explore the expected precision obtained from the planned study, the distance from the mean group difference of the primary endpoint (change from baseline in Chalder Fatigue Scale) to the limits is calculated using its 95% confidence interval (CI) and a common standard deviation of 5.8 as provided for patient with ME/CFS [23]. Using these assumptions and the given group sample sizes of 44 patients and 22 patients, the distance from mean to the limits of the 95% CI is 3.03 points in the Chalder Fatigue Scale. Assuming an expected drop-out rate of 20% (resulting in expected group sample sizes of 35 and 18 patients, respectively), the distance from the mean to the limits of the 95% CI increases to 3.37 points in the Chalder Fatigue Scale. The sample size calculation was conducted using PASS, Version 22.0.2.

### Recruitment {15}

Based on a recent publication, 45.2% of the patients with fatigue fulfil the diagnostic criteria for ME/CFS [6]. Patients with CFS, including PACS-CFS, who already have been admitted (approx. 600 within the last 18 months) demonstrate a high interest in participation in interventional studies to have the chance of possible effective treatment and to contribute to fostering the body of evidence on CFS. To screen the estimated 150 patients necessary for a final enrolment of 66 participants, we will screen at least 15 patients per month, a completely feasible number given the high prevalence of post-COVID-19 fatigue, the aforementioned high association with ME/CFS, and the high concentration of precisely these patients coming from all over Germany to our specialized outpatient centers.

## Assignment of interventions: allocation

### Sequence generation {16a}

A full randomization list of a 2:1 randomization (IA versus sham-IA) will be prepared using a validated computer-generated system. A blinded version of the randomization list will be imported into the electronic case report form (eCRF). Subsequently, eligible subjects will be assigned randomization numbers in ascending, sequential order from the imported list. Block randomization, stratified by PACS-CFS (i.e., if the patient suffers from PACS-CFS or not), will be applied to ensure comparable treatment groups within the stratum. Block lengths will vary to reduce the predictability of treatment allocations.

### Concealment mechanism {16b}

For each eligible patient, the study software will assign a unique identification (ID) independent of the enrollment center and link it to the patient’s pseudonym. The ID will also be shared with an unblinded member of the medical staff responsible for preparing the apheresis units based on an unblinded re-identification list. Every center receives a box with the full set of emergency envelopes. The unblinded re-identification list will be kept securely and confidentially. Only personnel not involved in the study and unblinded doctors and nurses from the nephrology department, who apply the treatment, will have access to the list.

### Implementation {16c}

Allocation sequence will be generated by the data management team, and this list will only be accessible to the data management team. A blinded staff member will not have access to the list. The investigators will enroll and document the patients in the eCRF. The unblinded team, responsible for preparing the apheresis units as mentioned above, will also assign participants to either IA or sham apheresis. We will implement regular training and clear standard operating procedures to ensure that all members of the nephrology department performing IA on patients do not disclose the treatment arm. Further study-related assessments will be only performed by the blinded team of neurologists.

## Assignment of interventions: blinding

### Who will be blinded {17a}

The study will be conducted in a double-blinded manner, meaning that both the study participants and the study team (including the investigator) except for study nurses and doctors from the nephrology department applying the treatment (operators of the LIFE21 apheresis unit) will remain blinded to the treatment arm until data analysis is completed to minimize any effects of bias on reporting of adverse events and effectiveness outcomes. During the IA, patients are in the Nephrology Clinic and are subsequently transported back to the Neurology ward. Due to this spatial separation, there is no risk of the study physicians being unintentionally unblinded.

### Procedure for unblinding if needed {17b}

In cases of safety issues, when emergency unblinding is expected to contribute to safety, emergency unblinding will be necessary and allowed. Each center is equipped with a box (identical triplets), containing individual letters for all possible randomizations. The study team can open the specific letter, tagged with the randomization ID, and will receive the information indicating whether the apheresis unit should be set up for sham or verum. The opaque, sealed envelopes contain twice-folded A4 sheet with, in addition to the ID, multiple layers of non-sensical words.

## Data collection and management

### Plans for assessment and collection of outcomes {18a}

Assessment and collection of screening, baseline, outcome, and other trial data will be performed by local investigators in the study visits. Every delegated assessor will be trained by a qualified trainer. We use the following study instruments: questionnaires, laboratory tests, neuropsychiatric examination, testing of hand grip strength, finger tapping test, 6MWT, and optional spirometry. A summary of the data to be collected is provided in Fig. 2.

### Plans to promote participant retention and complete follow-up {18b}

To promote participant retention and complete follow-up, patients will be very well supervised by our outpatient clinics so that with their support we promote motivation and readiness even after the therapy has been completed. We will also educate patients on the importance of follow-up visits to check the duration of possible clinical improvement with IA therapy. Besides, they will always have the chance to contact a responsible person at the site. Contact data (telephone numbers, email) are included in the informed consent form. Regardless of the reason for discontinuation, patients should be asked to return to the study center for follow-up assessments for safety reasons until 3 weeks after the last IA cycle unless consent is withdrawn. Post-treatment follow-up of each participant is of critical importance and is essential to preserving patient safety and the integrity of the study. Patients who withdraw early or were discontinued from study treatment must continue to be followed for collection of outcome follow-up data as in line with the required assessments listed in Fig. 2 until study completion. A patient should not be considered as lost to follow-up until careful verification has been completed: for patients whose status is unclear because they fail to appear for study visits without stating an intention to discontinue or withdraw, the investigator should make every effort to re-contact the patient to document his/her health status in the source documents. Attempts taken to contact the patient, e.g., dates of telephone calls, registered letters, etc., must be documented in the patient’s record.

### Data management {19}

Data management will be performed according to the data management plan. For each subject screened, an electronic case report form (eCRF) will be completed within the Electronic Data Capture (EDC) system and approved by the investigator. All data entered in the eCRF will be verifiable in source documentation other than the eCRF. Source documents will be filed at the study site according to local standard operating procedure (SOP). Trained study site staff will be responsible for entering subject source data into the validated EDC system. Data captured electronically will be immediately saved to the applicable database and changes tracked to provide an audit trail. Data validation procedures will be applied by data management to each stage of data handling to ensure that all data are reliable and have been processed correctly. Adverse events and medical/surgical history will be classified according to the terminology of the latest version of MedDRA. Medications will be classified according to the WHO Drug Dictionary. CRFs will be supplied for recording all study data from each patient. It is the responsibility of the investigator to ensure that the CRFs are completed in full and that the data therein are supported by source documentation. The CRFs must be kept in order and up to date so that they always reflect the latest observations on the patient. CRFs will be completed for each patient screened in the study. The study termination form of the CRF will be signed by the investigator at the end of the study confirming that he is satisfied with its completion and accuracy.

### Confidentiality {27}

Personal data are protected against unauthorized access, disclosure, and dissemination by technical and organizational measures. The study data are separated from the identifying data and the study data are pseudonymised. Re-identification is only to be carried out by the including trial site if is necessary, e.g., to assert the rights of the data subjects. Personal data and samples are always disseminated pseudonymously and only to the defined recipient (e.g., sponsor, laboratory).

### Plans for collection, laboratory evaluation, and storage of biological specimens for genetic or molecular analysis in this trial/future use {33}

Sample storage and management will be performed by different laboratories, including Labor Berlin – Charité Vivantes GmbH, Klinisch-immunologisches Labor Prof. Dr. med. Winfried Stöcker and CellTrend GmbH, Experimentelle Neurologie AG Prüß Charité Universitätsmedizin Berlin. Additionally, voluntary donations of biological (blood and CSF) samples may be collected for future investigations, including genetic research. The collected biological samples and related data are stored by the sponsor in a biobank, and they are made available for research purposes to enhance the prevention, detection, and treatment of diseases.

## Statistical methods

### Statistical methods for primary and secondary outcomes {20a}

Categorical endpoints will be summarized with absolute and relative frequencies by treatment group. Continuous endpoints will be summarized by treatment group using descriptive statistics (number of non-missing values, mean, median, standard deviation, minimum, and maximum) of the values at each visit and the change from baseline to each visit. The analysis of the primary endpoint will be based on all patients that were randomized and underwent at least one IA cycle (active or sham IA intervention). A linear regression modeling the change from baseline to 2 months of the Chalder Fatigue Scale score will be used. The treatment and subgroup allocation as well as age will be included as covariates to adjust for age and to investigate treatment and subgroup effects. The 95% confidence interval (CI) for treatment group difference will be based on least squares means. Additionally, a two-sample two-sided *t*-test will be conducted to investigate a potential treatment difference. Secondary endpoints related to the clinical effectiveness will be analyzed using descriptive methods. Exploratory statistical tests will be based on the underlying distribution of the respective endpoints and will be performed to investigate possible treatment differences. Resulting 95% CIs of the treatment differences will be provided, if possible and meaningful, and will be interpreted in an exploratory way. Since the analyses of the primary and secondary endpoints are all exploratory, the significance level of each test is 0.05, i.e., no correction for multiplicity will be applied. Data analysis will be performed by SAS® version 9.4 or higher. A full description of the effectiveness analyses can be found in the separated statistical analysis plan (SAP), which will be finalized prior to the unblinding of treatment allocation codes/database.

### Interim analyses {21b}

N.A. An interim analyses is not planned.

### Methods for additional analyses (e.g., subgroup analyses) {20b}

The following subgroups are defined: the PACS-CFS subgroup, including patients with post-acute COVID-19 CFS and CFS patients without post-acute COVID-19 CFS at randomization. Analyses of effectiveness endpoints in both subgroups will be exploratory.

### Methods in analysis to handle protocol non-adherence and any statistical methods to handle missing data {20c}

Data from patients who prematurely terminate the trial will be used up to the maximum extent possible. Patients who withdraw prior to the randomization can be replaced. For the main analysis, the data will be used as available. Imputation of missing data is intended as sensitivity analysis and will be specified in the SAP. Deviations from the protocol will be classified into “critical” and “non-critical” according to the potential impact on the statistical analysis.

### Plans to give access to the full protocol, participant-level data, and statistical code {31c}

The study protocol, the informed consent forms, participant-level dataset, and the statistical analysis plan will be made available on demand.

## Oversight and monitoring

### Composition of the coordinating center and trial steering committee {5d}

Information about the coordinating center is not applicable, because all participating sights belong to Charité Universitätsmedizin Berlin. The NKSG Steering Committee Platform (“National Clinical Study Group” Post-COVID Syndrome und ME/CFS), initiated at Charité Universitätsmedizin Berlin, will review the clinical trial. The steering committee includes members of the Charité COVID Research Board, as well as representatives of two patient organizations (ME/CFS group German Association for ME/CFS (DG ME/CFS) and the patient organization Long-COVID Deutschland (LCD)), who will give advice to this network. Moreover, members of the NUM NAPKON-TIP participate in this steering committee to facilitate immediate translation of promising clinical results in future multicenter trials. Safety data will be regularly reviewed by the study coordinator. In case of an unexpected high number of safety issues, data will be discussed within the steering committee of the NKSG. Data will be monitored on a regular basis by the Clinical Trial Organization (CTO) of the NKSG. There will be also data monitoring of final data by the CTO of the NKSG. Study progress will be regularly presented to the steering committee.

### Composition of the data monitoring committee, its role and reporting structure {21a}

The study is an investigator-initiated study, conducted by the Department of Neurology at Charité Universitätsmedizin Berlin. The Charité will be the sponsor. This is a medical device study with no dose escalation or switch to multiple dosing. The intervention device and all procedures are well-described and approved by Health Authorities worldwide for other indications. It cannot be assumed that patient safety is dependent on the indication. Nevertheless, a data and safety monitoring board (DSMB) will monitor the data for safety and tolerability of each study participant from screening until completion of study his/her participation. The DSMB consists of three physicians with high expertise in neurology and endocrinology. All DSMB members are experts in clinical aspects of their field as well as clinical trial conduct and methodology. The chosen DSMB members are neither representatives of the sponsor or of the manufacturer of the investigational medicinal product nor any other individual with vested interests in the outcome of the study. The responsibilities of the DSMB include reviewing participant safety data, assessing study progress and protocol adherence, and proving recommendations to the sponsor and principial investigator on study continuation, modification, or termination. The frequency of DSMB meetings will be determined by factors such as enrollment rate, safety issues, adverse events, and data availability. The chair of the DSMB will define the meeting schedules, with the first meeting held after ten patients have completed 5 IA cycles. A written summary report will be issued after each meeting, detailing topics discussed, findings, safety assessment, and recommendations. The DSMB will monitor participant safety throughout the trial, including the review of adverse events reports, evaluation of other relevant safety data, and assessment of participant recruitment and retention rates. In addition, safety measures will be clearly defined in the study protocol by introducing stopping rules for individual subjects, cohorts, and the entire study.

### Adverse event reporting and harms {22}

The sponsor has established standard operating procedures in conformity with the applicable regulatory requirements laid down in the MDCG 2020-10/1 (Safety reporting in clinical investigations of medical devices under the Regulation (EU) 2017/745) to ensure appropriate reporting of safety information of this clinical study in accordance with those procedures.

### Frequency and plans for auditing trial conduct {23}

A quality assurance audit/inspection of this study may be conducted by the sponsor, sponsor’s designees, or by independent ethics (IEC) or by regulatory authorities. The quality assurance auditor will have access to all medical records, the investigator’s trial-related files and correspondence, and the informed consent documentation of this clinical study.

### Plans for communicating important protocol amendments to relevant parties (e.g., trial participants, ethical committees) {25}

Any significant change in the study requirements, design, or scheduled activities requires a protocol amendment to be issued. The investigator will not make any changes to the study or deviate from this protocol without competent regulatory authority(s), ethical committee, and sponsor approval, except when necessary to avoid immediate danger to patients.

### Dissemination plans {31a}

The study results will be published in a peer-reviewed international journal. To maximize the project impact and allow perfect dissemination of project results and knowledge transfer with the scientific community, scientific achievements of this trial will be published and presented in high impact journals and relevant international congresses, making the obtained research data findable, accessible, interoperable, and reusable for researchers all over the world (with the objective to generate FAIR datasets). The principle of open access to peer-reviewed scientific publications will ensure free access for the widest possible readership in these research areas.

## Discussion

The etiology and the mechanisms leading to ME/CFS are largely unknown, as is the pathogenesis of CFS related to long-COVID. In a subgroup of patients, we assume that an overactivation of the immune system may lead to hyperinflammation and autoantibody production. Autoantibodies (against various antigens, including neurotransmitter receptors) have been identified in several cohorts of patients with post-infectious ME/CFS. Due to the broad antibody spectrum, specifying relative frequencies is only possible to a limited extent. The more intensely studied neurotransmitter receptors belong to the group of G protein-coupled receptors (GPCRs). With respect to autoantibodies against GPCRs, they were found in up to 30% of patients with ME/CFS [7]. Autoantibodies against individual GPCRs such as ß2-adrenoreceptors and α1-adrenoreceptors, nociceptors, and angiotensin II-, muscarinic M_2_-, MAS-, and ET_A_-receptors were also found in sera from patients with long-COVID symptoms with neurological and/or cardiological origin [14]. Moreover, these autoantibodies correlated with symptom severity (e.g., fatigue, vasomotor, and cognitive symptoms) in patients with PACS-CFS [31]. Recently, one study showed that autoantibodies targeting brain epitopes were common in patients with post-COVID syndrome (PCS) and strongly associated with pathological cognitive screening test, especially when they were detected in CSF [32]. These studies also demonstrated that, in addition to the large clinical overlap, many commonalities may exist at the antibody level, emphasizing the need to study both patient populations: ME/CFS with PACS-CFS. However, as this is a very recent development in clinical medicine, the epitopes and nature of the putative autoantibodies are not fully characterized. Therefore, it would not make sense to select a single (or a small panel of) autoantibody for the present study as an inclusion criterion but have the detection of any type of COVID-19/ME-CFS-associated autoantibodies for a person to qualify for this study. A major advantage of this strategy is that this trial could help not only to understand therapeutic effectiveness but also to gain knowledge towards the pathophysiological basis of the disease. In addition, a recent observational study showed that half of the PCS patients with moderate to severe fatigue and exertional intolerance fulfil the CCC 2003 for ME/CFS [33]. Until now, there are no therapeutic options to cure ME/CFS, although it causes an immense individual, social, and economic burden. Preliminary data from several single-center projects treating patients with the same indication and the same therapeutic platform reveal a rate of partial or complete remission above 50% of participants, based on oral communication or congress presentations. There is a high need for large, randomized, and placebo-controlled trials to replace controversial experimental therapeutic approaches by evidence-based decision making in the future.

In this study, a control group, receiving a sham IA via a jugular or peripheral venous line is included. Sham IA is justified in this early phase, as it will minimize the potential bias in reporting adverse events, the assessment of outcome measurements, and assessment of immunoglobulin levels and autoantibodies during this study. Most importantly, patients undergoing invasive measures such as IA regularly experience a relatively strong placebo effect. Without a sham group, this strong placebo effect could result in the interpretation of false-positive effects, which may lead to approval/recommendation of the procedure despite lack of effectiveness. As a result, the comparatively high individual impact for the study participants to receive a possible sham procedure is strongly justified by the need for placebo-controlled evidence for future therapeutic options and has already been safely employed for plasma exchange [34]. The validated, broadly employed, and simple to use Chalder Fatigue Scale was chosen as primary endpoint because it offers huge advantages, including high reproducibility, comparability, and reliability. The lack of a simple score reflecting the complexity of symptoms caused by ME/CFS will be compensated by diverse secondary endpoints which are specifically designed to profoundly characterize the disease, understand its etiology, and discover biomarkers to assure reliable diagnostic processes in the future. The results of this study therefore may not only offer an important contribution to the establishment of innovative evidence-based treatment options and to the later identification of suitable patients in clinical routine but also to the etiological understanding and therefore establishment of reliable diagnostic tools for ME/CFS.

## Trial status

The conductance of the study has been approved by the responsible local ethic committee. At the beginning of the study, protocol version number 3.1 Including Amendment 3/JUN 2023 is used. The beginning of recruitment is planned for 15 October 2023; end of recruitment is estimated for 15 October 2024.

## Abbreviations

6MWT: 6 Minute walking test
ACE: Angiotensin-converting enzyme
AE: Adverse event
BL: Baseline
CCC: Canadian consensus criteria
CFS: Chronic fatigue syndrome
CI: Confidence interval
cMRI: Cranial magnetic resonance imaging
CMV: Cytomegalovirus
CRO: Clinical research organisation
CSF: Cerebrospinal fluid
CTO: Clinical trial organization
DSMB: Data safety and monitoring board
EBV: Epstein-barr virus
ECG: Electrocardiography
eCRF: Electronic case report form
EDC: Electronic data capture
EOS: End of study visit
EOT: End-of-treatment visit
ET: Endothelin
HBV: Hepatitis B virus
HCV: Hepatitis C virus
HIV: Human immunodeficiency virus
IA: Immunoadsorption
ID: Identification
IEC: Independent ethics
Ig: Immunoglobulin
IVIG: Intravenous immunoglobulin
LCD: Long-Covid Deutschland
LVEF: Left ventricular ejection fraction
MAR: Muscarinic acetylcholine receptors
MAS: Metabolite angiotensin
ME/CFS: Myalgic encephalomyelitis/Chronic fatigue syndrome
MoCA: Montreal-cognitive-assessment-test
NKSG: Nationale Klinische Studiengruppe
PACS-CFS: Post-acute COVID-19 CFS
PCFS-Scale: Post-COVID-19 functional status scale
PEM: Post-exertional malaise
PROMIS: Patient-reported outcomes measurement information system
QoL: Quality of life
RCTs: Randomized controlled trials
SAEs: Serious adverse events
SAP: Statistical analysis plan
SDMT: Symbol digit modalities test
SOP: Standard operating procedures
SUSAR: Suspected unexpected serious adverse reaction
TBC: Tuberculosis
TEAEs: Treatment emergent adverse events
TPO: Thyreoperoxidase
WHO: World health organization
ß2AR: Beta-2-adrenergic receptors
